# Implant-Associated Infections: A Review of the Safety of Cardiac Implants

**DOI:** 10.7759/cureus.12267

**Published:** 2020-12-25

**Authors:** Venkataramana Kandi, Sabitha Vadakedath

**Affiliations:** 1 Clinical Microbiology, Prathima Institute of Medical Sciences, Karimnagar, IND; 2 Biochemistry, Prathima Institute of Medical Sciences, Karimnagar, IND

**Keywords:** cardiac implantations, rhythm of the heart, conduction, failure of implants, implant associated infections, etiology, predisposing factors, preventive strategies

## Abstract

Cardiac implantations are among the most critical, and life-saving patient management procedures. Most cardiac implantations are performed to correct abnormalities in the conduction and the rhythm of the heart. Because the implants are intended for long-term use ranging from months to years, the failure of an implant is considered a major setback both in the patients as well as surgeons’ perspectives. Implant failures can have multifactorial reasons, amongst which infectious causes need to be adequately addressed. This review attempts to evaluate the nature of implants, etiology, predisposing factors, infection control, and preventive strategies for cardiac implant-associated infections.

## Introduction and background

Infection is a result of the invasion of microorganisms into the host. The infections are either endogenous or exogenous, wherein the exogenous infections occur because of the entry of microbes into the humans by various modes that include inhalation, ingestion, contact, inoculation, and from mother to the child. The endogenous infections develop in humans due to the invasion and proliferation of microbial species that are already present in the human body as commensals. Although most microbial invasions do not lead to infection, the consequence of a microbial invasion depends on several factors. The implant-associated infections (IAIs) are infections associated with the implants which are generally used to treat/manage patients. The implants are made up of metals, and other synthetic materials that may be used as a substitute, to support and balance while managing patients suffering from various conditions that include cardiac conduction abnormalities, valve dysfunctions, bone fractures, and others. The IAIs depend on the nature of the implant, the type of procedure, the experience of the surgeon, virulence determinants of the invading microbe, and the status of the host's immune system influence the IAIs. The present review delineates the nature of implants, IAIs, cardiac IAIs, etiopathogenesis, predisposing factors, control, and preventive strategies to minimize IAIs.

## Review

Implants

Implants are devices used by health professionals during patient management. An implant may be any device that is incorporated into the human body, usually temporarily, as done in the case of catheters being inserted in the patient management for various conditions. The implants may be metal devices that are used for support by the orthopedic surgeons during a bone repair/corrective surgery. Also, the dental surgeons use various types of metal, and non-metal antibiotic-impregnated and non-impregnated implants during different types of teeth, oral and maxillofacial surgeries.

An implant could be a prosthetic device that is majorly used as a support, barrier, or a substitute for a human organ like the bone, joint, breast, hip, pelvis, mitral valve, teeth, cardiac stents, biliary stents, and others. Also, implants (mesh-like, rubber, synthetic, and plastic) are available to treat various conditions that include the collapse of pelvic organs and hernias [1-3].

Conversely, an implant may be an electronic device used for the proper functioning of muscles, brain, and heart. Cardiac implants that are frequently used to treat heart patients including cardiac implantable electronic devices (CIED), such as a pacemaker, implantable cardioverter-defibrillator (ICD), implantable cardiac loop recorder, and cardiac resynchronization therapy devices [4,5].

Implants for human use may be made up of metal, plastic, rubber, silicone, graphene, or animal tissue (pig tissue) [6]. The metals which are rust-free like stainless steel, titanium, gold, silver, platinum, and tungsten, and other alloy-based metals, and liquid amalgams including the chromium-cobalt, and nickel-titanium are used to prepare the implants [7].

In recent research findings, the use of polymer-zinc oxide nanoparticle composite films in the preparation of cardiac implants that function as electronic devices is recommended. This material was found to have improved insulating capabilities, and do not react or interfere either with other host biofluids/environment and electrically active human tissues like the brain and spinal cord [8].

The major concern arising from the presence of an implant in human beings is the allergic/hypersensitive reaction and inflammatory response that may be mounted by the host's immune system against the implant/device [6].

Since an implant remains for a long period ranging from a few months to several years, the occurrence of IAIs and their potential to cause severe morbidity is often a cause for serious concern [9]. There are several reports of IAIs resulting in severe morbidity and mortality [10,11]

 Etiopathogenesis

**Figure 1:**
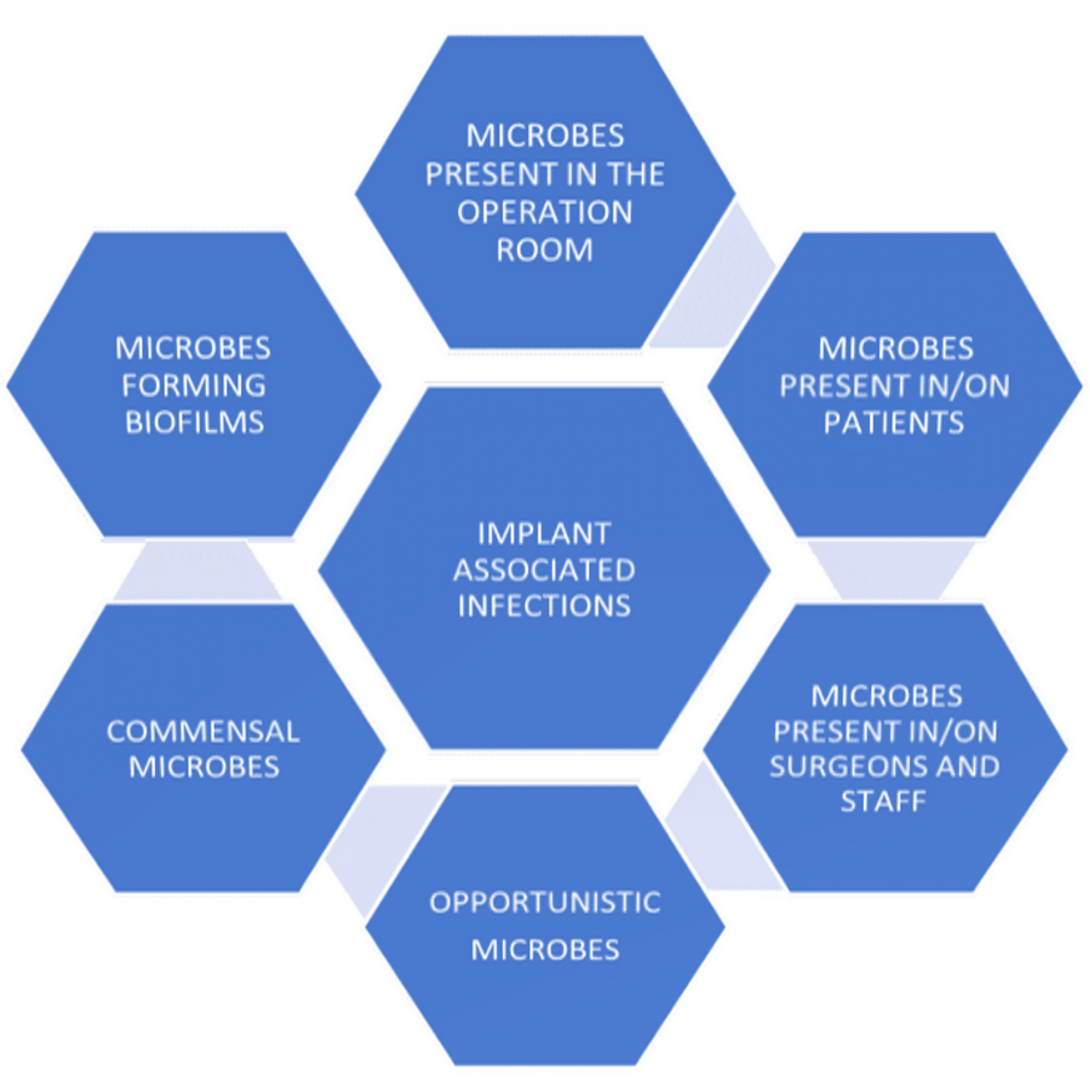
The microbial origins and characteristics that facilitate IAIs IAIs: implant-associated infections

The implants act as foreign surfaces in the human body, thereby facilitating the colonization of microbial species, that hitherto is cleared by the host's innate immune mechanisms. The reason for this is the fact that the bacteria that adhere to such implant surfaces are less susceptible to killing/elimination by phagocytosis. Also, the bacteria survive on the implant surfaces and develop biofilms that reduce the effect of antimicrobial agents and result in persistent colonization [12]. Bacteria that form biofilms were noted to demonstrate increased antimicrobial resistance and infections by such microbes are difficult to treat [13]. The microbial origins and the characteristics that facilitate IAIs are shown in Figure 1.

The adherence of bacteria to the implant surfaces is facilitated by the presence of fimbriae, and surface proteins like fibronectin, fibrinogen, and collagen-binding proteins, and microbial surface components recognizing adhesive matrix molecules (MSCRAMM) [14,15].

The microbes including the *Staphylococcus *species (sp.), *Streptococcus *sp., *Corynebacterium *sp., *Propionibacterium acnes *(Gram-positive), *Escherichia coli*, *Pseudomonas *sp., *Klebsiella pneumoniae*, *Providentia stuartii *(Gram-negative), some fungi, and *Mycobacterium *sp. can form biofilms and thereby pose an increased risk of IAIs [16,17]. Previous studies have noted that cardiac implants could pose an increased risk of infections with up to 40% of ventricular assisted device (VAD) patients developing implant-related infections [18,19].

These bacteria which form biofilms on the implants elicit almost continuous inflammatory responses, thereby creating an environment that potentially causes failure of the implant [20].

An evaluation of stent associated respiratory tract infections (SARTI) among patients with stent implantation revealed that *S*. *aureus *(50%) and *Pseudomonas aeruginosa *(35.7%) were predominant pathogens followed by *Candida* *albicans *[21]. Infective endocarditis caused by Gram-negative bacteria like *E*. *coli*, *P*. *aeruginosa*, and *K*. *pneumoniae*, were frequent among cardiac implant patients with immunosuppression and chronic genitourinary colonization of bacteria [22].

Methicillin-resistant *S*. *aureus *(MRSA) (23.4%) was the most frequent cause of cardiac permanent pacemaker associated infection followed by Methicillin sensitive *S*. *aureus *(14.9%), *Pseudomonas *(10.6%), *E*. *coli *(8.5%), and *Klebsiella *(6.4%) [23]. *S*. *aureus* and *S*. *epidermidis *were the two frequently isolated bacteria associated with cardiac IAIs among patients who were implanted with both new and re-used pacemakers and defibrillators [24]. 

The occurrence of microbes having the ability to grow slowly and cause persistent infections which are difficult to treat by traditional antimicrobial therapy regimens may pose an increased threat in debilitated patients that include patients with cardiac implants. Such variants of microbes are referred to as small colony variants (SCVs). Interestingly, most common IAI causing microbes like *S*. *aureus*, *E*. *coli*, and *Pseudomonas *sp. have been noted to form SCVs [25].

Most microbial species that are frequently associated with IAIs can produce slime, and capsules which help them to adhere to host cells, and implant surfaces thereby evade inhibition and neutralization by phagocytosis, complement-mediated lysis, and other immunological responses. Also, such microbial species resist killing by antimicrobial therapeutic agents [26]. 

Predisposing factors

**Figure 2:**
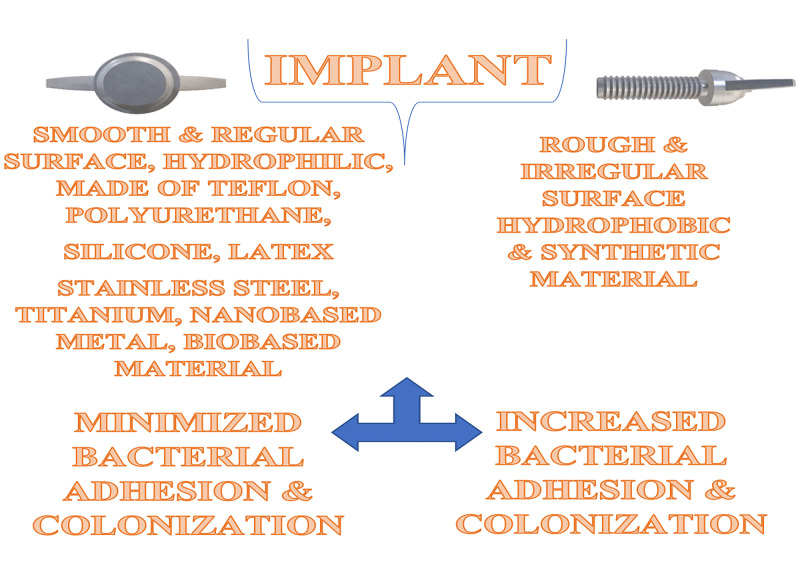
The characteristics of implants

The IAIs form an integral part of hospital-acquired/associated infections (HAIs) [27]. Among the various factors that predispose to IAIs, the nature of the implant assumes increased significance. Implants are made of polyvinyl chloride, polyethylene, latex, silicone, and stainless steel material. Implants with a rough texture and irregular surfaces favor bacterial colonization as compared to the smooth and hydrophilic implants [10]. Synthetic materials have been noted to encourage bacterial adherence and colonization as compared to biomaterials as shown in Figure 2.

Factors that predispose cardiac implant patients to infections include extremes of age, presence of co-morbidities like diabetes mellitus, congestive heart failure, impaired renal function, malignancy, use of immunosuppressive drugs, corticosteroids, and anticoagulants [28,29].

A recent study that evaluated the frequency, etiology, and risk factors for left ventricular assist device (LVAD)-associated infections found that more than 30% of patients developed IAIs. The most frequently isolated bacterium was *S*. *aureus *(45.4%), followed by the *Enterobacteriaceae *members (24.6%), *P. aeruginosa *(13.7%), and coagulase-negative *Staphylococcus *(CONS) (5.2%). About 5.2% of patients had reportedly suffered IAI caused by *Candida *sp. Increased age (>58 years) and the type of implant were considered as the risk factors for IAIs [30].

Predisposing factors related to the CIED related infections were systematically reviewed in a previous study. This study had noted that the IAIs could be associated with the host factors, the operative procedures (pre-, peri-, and post-operative), and the implantable device factors. The host factors that predispose to IAIs include diabetes mellitus, end-stage renal disease (ESRD), chronic obstructive pulmonary disease, corticosteroid and anticoagulant therapy, history of a previous device-associated infection, heart failure, renal insufficiency, pre-surgical fever episodes, and skin disorders. The procedure-related causes for IAIs included the inexperienced surgeon, implant replacement/revision procedures, lack of adequate antibiotic therapy prior, and during the procedures [31].

The procedural perforation of the right ventricle in patients who were undergoing pacemaker implantation that leads to pericardial effusion and cardiac tamponade was recently reported [32,33]. These case studies highlight the role of procedural complications and the role of iatrogens/surgeons in the development of IAIs.

**Figure 3:**
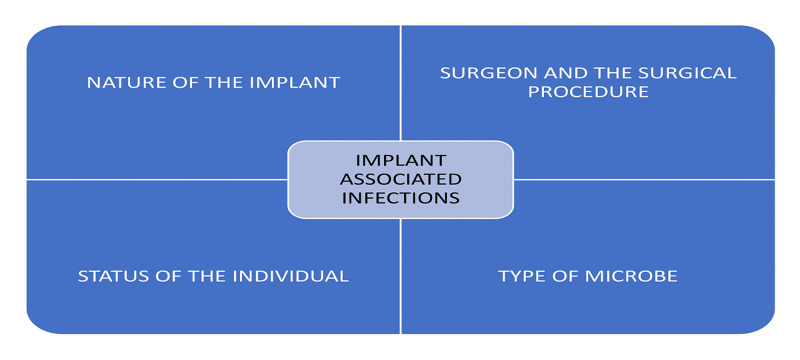
The causes of IAIs IAIs: implant-associated infections

In a recent study, the presence of pocket hematoma was significantly associated with wound and cardiac IAIs [34]. The IAIs may be associated with the patient factors, the procedural and personnel issues, the microbial factors, and the nature of the implant as shown in Figure 3.

Complications arising from cardiac implants were assessed in a previous study. Majorly, the implant-associated technical failures, loosening/dislodging of the implants from the site, pocket erosion, and other adverse clinical events like cardiac tamponade, perforation, hematomas, pneumo/hemothorax, and thrombotic, and thromboembolic consequences were noted in cardiac implant patients. Also, it was noted that infection was another significant complication associated with cardiac implantations as evidenced by the occurrence of pain, peripheral nerve injury, phlebitis, and cellulitis [35].

Infection control and preventive measures 

Because the implants may be colonized by the microbes with the help of biofilms, a previous study had reported the benefits of an alloy silver-coated titanium-aluminum-niobium (TiAlNb) as an implant material that could potentially minimize the colonization and thereby reduce the rates of IAIs. This study had also observed that the implants coated with antibiotics like vancomycin and daptomycin minimized the colonization of multi-drug resistant (MDR) bacteria like the MRSA [36].

Several pre-, peri-, and post-operative procedures are recommended to minimize the incidences of IAIs among cardiac implant surgical patients. Systemic antibiotic prophylaxis, a thorough screening of patients for remote infections, preoperative antiseptic shower, decontamination of skin at the operation/incision site, and use of protective barriers like gloves, and masks were recommended to prevent implant-related post-surgical complications [28].

The efficiency of liquid-infused surface materials when coated on the implants and other medical devices minimized the microbial adhesion and colonization. Such materials are also found efficient for drug delivery [37].

The European Heart Rhythm Association (EHRA) has recently collaborated with the European Association for Cardio-Thoracic Surgery (EACTS) and framed an international consensus document that is endorsed by the Heart Rhythm Society (HRS), the Asia Pacific Heart Rhythm Society (APHRS), the Latin American Heart Rhythm Society (LAHRS), International Society for Cardiovascular Infectious Diseases (ISCVID) and the European Society of Clinical Microbiology and Infectious Diseases (ESCMID). This document incorporates elaborate guidelines and suggestions to prevent, diagnose, and treat IAIs. It emphasizes the need for continuous monitoring of implant patients by maintaining a registry both at the national and international levels. Also, this document recommends research evidence for the use of antibiotic coated implants in minimizing infections and implant failures, duration of postoperative antibiotic therapy, appropriate timing for reimplantation surgeries, and the long term risk of death due to implant infection-related and other complications [38].

The comprehensive bundle approach that recommends the use of novel minocycline and rifampicin antimicrobial mesh (TYRX) to prevent the IAIs in patients suffering from both solid organ and hematologic malignancies with CIED was reported recently [39]. Such an approach was previously confirmed as an important strategy to prevent cardiac IAIs as evidenced by the results of the Worldwide Randomized Antibiotic Envelope Infection Prevention Trial (WRAP-IT). This trial recommends the use of TYRX, an antibiotic eluting mechanism that releases the antimicrobial agent into the surgical pocket for a minimum of seven days and thereby prevents IAIs among high-risk patients including cancer patients [40]

Miniaturized and micro implantable devices used to treat cardiac conditions were noted to substantially minimize the risk of complications and infection-related hospitalizations among patients [5].

Currently, no evidence supports the prescription of antibiotics after implant surgery. A recent study had attempted to evaluate the patients who have been prescribed both presurgical and postsurgical antibiotics after cardiac implant surgery. This study had found no significant difference of susceptibility to infections in patients who were given presurgical antibiotic course and those who received both presurgical and postsurgical antibiotic therapy. Also, it was suggested that postsurgical antibiotics must be prescribed only after considering patient factors that include age, presence of co-morbidities, and others [41].

A more than two-fold increased risk of acquiring infections is associated with the cardiac device implantation, replacement, and repair surgeries. It is important to identify patients at risk, diagnose the infection using pathogen detection in blood cultures, and finding pathogen biofilms by imaging techniques and initiate appropriate and specific antimicrobial therapy to minimize the morbidity and mortality associated with IAIs [42-44].

Strict adherence to infection control procedures during implantation surgeries could prevent the contamination of implant by skin flora and the use of antimicrobial enveloped implants minimize the chances of IAIs [45].

A recent study had suggested that although several factors including the patient, procedural, and others that contribute to IAIs, the use of anticoagulant and antiplatelet therapy could reduce the risk of cardiac IAIs [46].

## Conclusions

The cause of IAIs seems to be multifactorial, wherein the patient factors, the surgeon, and procedural causes, the nature of the implant, the source, type, and the ability of the microbes may all influence the occurrence of IAIs. The IAIs could be controlled and minimized by using improved implant materials that include nano-based materials, biobased implants, leadless implants, and implants precoated with antibiotics. Also, the IAIs may be prevented by thoroughly screening the patients and operation theatre staff for microbes colonized in the mucosal surfaces and the skin, clinically evaluating the patients for prior bacteremia, fever episodes, and potential co-morbidities prior to implantation procedures, and timely initiation of preprocedural antibiotic therapy. 
